# Characterization of the quinolone resistant determining regions in clinical isolates of pneumococci collected in Canada

**DOI:** 10.1186/1476-0711-9-3

**Published:** 2010-01-18

**Authors:** Samir N Patel, Roberto Melano, Allison McGeer, Karen Green, Donald E Low

**Affiliations:** 1 Department of Microbiology, Mount Sinai Hospital, Toronto, Ontario, M5G 1X5, Canada; 2 Department of Laboratory Medicine and Pathobiology, University of Toronto, Toronto, Ontario, M5G 1L5, Canada; 3 Ontario Agency for Health Protection and Promotion, Toronto, Ontario, M9P 3T1, Canada

## Abstract

**Background:**

The objective of this study was to examine *Streptococcus pneumoniae *isolates collected from a longitudinal surveillance program in order to determine their susceptibility to currently used fluoroquinolones and of the frequency and type of mutations in the quinolone-resistant determining regions (QRDRs) of their *parC *and *gyrA *genes.

**Methods:**

The Canadian Bacterial Surveillance Network has been collecting clinical isolates of *S. pneumoniae *from across Canada since 1988. Broth microdilution susceptibility testing was carried out according to the Clinical and Laboratory Standards Institute guidelines. The QRDRs of the *parC *and *gyrA *genes were sequenced for all isolates with ciprofloxacin MIC ≥ 4 mg/L, and a large representative sample of isolates (N = 4,243) with MIC ≤ 2 mg/L.

**Results:**

A total of 4,798 out of 30,111 isolates collected from 1988, and 1993 to 2007 were studied. Of those isolates that were successfully sequenced, 184 out of 1,032 with mutations in *parC *only, 11 out of 30 with mutations in *gyrA *only, and 292 out of 298 with mutations in *parC *and *gyrA *were considered resistant to ciprofloxacin (MIC ≥ 4 mg/L). The most common substitutions in the *parC *were at positions 137 (n = 722), 79 (n = 209), and 83 (n = 56), of which substitutions at positions 79 and 83 were associated with 4-fold increase in MIC to ciprofloxacin, whereas substitutions at position 137 had minimal effect on the ciprofloxacin MIC. A total of 400 out of 622 isolates with Lys-137 *parC *mutation belonged to serotypes 1, 12, 31, 7A, 9V, 9N and 9L, whereas only 49 out of 3064 isolates with no mutations belonged to these serotypes. Twenty-one out of 30 isolates with substitutions at position 81 of the *gyrA *gene had an increased MIC to ciprofloxacin. Finally, we found that isolates with mutations in both *parC *and *gyrA *were significantly associated with increased MIC to fluoroquinolones.

**Conclusions:**

Not all mutations, most frequently Lys-137, found in the QRDRs of the *parC *gene of *S. pneumoniae *is associated with an increased MIC to fluoroquinolones. The high prevalence of Lys-137 appears to be due to its frequent occurrence in common serotypes.

## Background

*Streptococcus pneumoniae *is a common cause of acute exacerbation of chronic bronchitis, sinusitis, and community-acquired pneumonia [1-3]. Treatment of respiratory tract infections has relied primarily on the use of β-lactam and macrolide antimicrobials; however in recent years the emergence of resistance to these agents has led to the widespread use of fluoroquinolones for adult patients with respiratory disease [4-7].

From the mid 1980s until the later part of the 1990s, earlier generation fluoroquinolones such as ciprofloxacin and ofloxacin were widely used for treatment of respiratory tract infections, despite having less than optimal activity against pneumococci. However, since the late 1990s, levofloxacin and moxifloxacin, with much greater *in vitro *activity and more optimal pharmacokinetic and pharmacodynamic properties for the treatment of pneumococcal infections, have replaced these agents [8,9].

Fluoroquinolones target one of the two type II topoisomerases of pneumococci, DNA gyrase (composed of GyrA and GyrB subunit) and/or topoisomerase IV (composed of ParC and ParE subunits), resulting in inhibition of DNA synthesis [10-12]. Decreased susceptibility of *S. pneumoniae *to the fluoroquinolones has been attributed to either chromosomal mutations in the quinolone resistance-determining regions (QRDRs) of the *parC *and/or *gyrA *genes, which encode for subunits of topoisomerase IV and DNA gyrase, respectively, and/or by a reserpine-sensitive efflux mechanism [13-16]. Fluoroquinolones have binding preferences (primacy) for one of the enzymes found in the two type II topoisomerases. Primacy is determined most reliably by *in vitro *selection experiments in which a resistance mutation is observed first in one of the two genes of the primary target and gene mutations in the secondary target are only observed subsequently [17]. Mutant selection studies in *S. pneumoniae *have shown that the primary target for fluoroquinolones, such as ciprofloxacin, ofloxacin and levofloxacin, is topoisomerase IV. In contrast, newer fluoroquinolones such as moxifloxacin, gatifloxacin and gemifloxacin primarily target DNA gyrase [18,19]. In fact, gemifloxacin and moxifloxacin have been found in some reports to exhibit dual activity based on the minimal effect of either *gyrA *or *parC *mutations on resistance, whereas strains with mutations in both *gyrA *and *parC *exhibit high-level resistance to these agents [20-22].

Previous studies have shown that pneumococci with substitutions at positions 79 and 83 of *parC*, and 81 and 85 of *gyrA *are most frequently found in isolates with reduced susceptibility to fluoroquinolones [23-32]. However, many of these studies only sequenced isolates that were resistant to fluoroquinolones, were smaller in sample size, and had been done in a time before the widespread use of the newer generation fluoroquinolones. The purpose of this study was to characterize a large collection of contemporary isolates of pneumococci in order to identify mutations in the QRDR of their *parC *and *gyrA *genes, and to determine the effect of these mutations on fluoroquinolone activity.

## Materials and methods

The Canadian Bacterial Surveillance Network is comprised of more than 100 private laboratories and community and university-affiliated hospitals in all 10 provinces and 2 of 3 territories in Canada. In 1988, and in each year from 1993 to 2008, participating laboratories were asked, based on their size, to collect either the first 20 (for small laboratories) or the first 100 (for larger laboratories) consecutive clinical isolates, and then all sterile site isolates of *S. pneumoniae*. The date of collection, the source of the specimen, and the patient's age and sex were recorded on a standard form. Duplicate isolates from the same patient were excluded. Isolates were transported on chocolate agar slants or swabs to a central laboratory where they were confirmed to be *S. pneumoniae *by standard methodology and stored at -70°C. Prior to susceptibility testing, isolates were thawed and subcultured onto blood agar twice. Isolates were serotyped on the basis of capsular polysaccharide antigens by the quelling reactions [33]. *In vitro *broth microdilution susceptibility testing was performed and interpreted according to Clinical and Laboratory Standards Institute (CLSI) guidelines [34,35]. Isolates were classified as non-susceptible to an antimicrobial if their MICs were in either the intermediate or resistant category, and resistant to ciprofloxacin if their MIC was ≥4 mg/L.

### Amplification and sequencing of QRDRs

The QRDRs of the *parC *and *gyrA *genes were amplified using following primers: for *parC *(forward: CGGTTCAACGCCGTATTCTT, reverse: ATCCCAGTCGAACCATTGAC; 394 bp), *gyrA *(forward: TGTTCACCGTCGCATTCTCT, reverse: ATACCAGTTGCTCCATTAACC, 393 bp). Amplicons were sequenced at Agencourt Bioscience Corp. (Beverly, MA). Multiple nucleotide sequences were performed with the ClustalW2 program http://www.ebi.ac.uk/Tools/clustalw2/index.html. Both *parC *and *gyrA *genes of isolates with a ciprofloxacin MIC ≥ 4 mg/L, a levofloxacin MIC ≥ 4 mg/L or a moxifloxacin MIC ≥ 0.5 mg/L were amplified and sequenced (N = 540). In addition, a 45% sample of isolates (N = 2,102) with a ciprofloxacin MIC = 2 mg/L, or a levofloxacin MIC of = 2 mg/L and or a moxifloxacin MIC = 0.25 mg/L, and a 12% sample of other isolates (N = 27,469) were also amplified and sequenced.

### Statistical Analysis

Differences in group proportions were assessed with the chi square test for trend or fisher's exact test.

## Results and Discussion

A total of 30,111 isolates were collected from 1988, and 1993 to 2007. Of these isolates, 11,403 (37.9%) were recovered from a sterile site (10,565 from blood, 371 from CSF, and 467 from other sterile sites), and 18,601 (61.8%) were from non-sterile sites (9,694 from sputum, 4,732 from eye, 1,988 from ear, 1,053 from bronchial washings, and 1,134 from other non-sterile sites). Overall, 534 out of 30,111 (1.8%) of isolates were resistant to ciprofloxacin (MIC ≥ 4 mg/L); 360 (1.2%) were non-susceptible to levofloxacin (MIC ≥ 4 mg/L), and 283 (1.1%) were non-susceptible to moxifloxacin (MIC ≥ 2 mg/L).

We selected 4,798 isolates for molecular characterization of *parC *and *gyrA *genes. Of these, 517 out of 534 isolates with ciprofloxacin MIC ≥ 4 mg/L, 968 out of 968 isolates with MIC = 2 mg/L, and 3,267 out of 3,296 isolates with MIC < 2 mg/L were successfully sequenced. As shown in Table 1, 1,032 isolates contained at least one mutation in *parC*, 30 isolates contained at least one mutation in *gyrA*, and 298 isolates contained at least one mutation in both *parC *and *gyrA*. Of those *parC *only mutant isolates, 17.8% were resistant to ciprofloxacin, 2.2% were non-susceptible to levofloxacin, and 0.4% to moxifloxacin. Among *gyrA *only mutant isolates, 36.7% were resistant to ciprofloxacin, 36.7% and 20% non-susceptible to levofloxacin and moxifloxacin, respectively. Finally, among isolates containing mutations in both *parC *and *gyrA*, 98.0% were resistant to ciprofloxacin, 97.6% non-susceptible to levofloxacin, and 86.2% to moxifloxacin (Table 1).

**Table 1:** *In vitro *activities of fluoroquinolones against *S. pneumoniae *isolates with mutations in the *parC and gyrA *genes.

		Minimum Inhibitory Concentration (mg/L) Number of Isolates														
		**0.015**	**0.03**	**0.06**	**0.12**	**0.25**	**0.5**	**1**	**2**	**4**	**8**	**16**	**32**	**64**	**128**	**Total**

***parC *only**																

Ciprofloxacin		0	0	0	0	2	182	408	256	136	39	5	2	2	0	1032

Levofloxacin		0	0	1	0	0	205	580	213	15	3	4	1	0	0	1022

Moxifloxacin		0	5	130	489	343	16	8	2	1	1	0	0	0	0	995

***gyrA *only**																

Ciprofloxacin		0	0	0	0	0	1	9	9	2	5	2	2	0	0	30

Levofloxacin		0	0	0	0	0	1	13	5	3	4	4	0	0	0	30

Moxifloxacin		0	0	1	6	8	4	5	4	2	0	0	0	0	0	30

***parC *& g*yrA***																

Ciprofloxacin		0	0	0	0	0	0	3	3	4	29	73	126	55	5	298

Levofloxacin		0	0	0	0	0	1	6	0	13	78	151	37	11	1	298

Moxifloxacin		0	0	0	4	6	3	26	136	87	18	1	1	0	0	282

**Figure 1:**
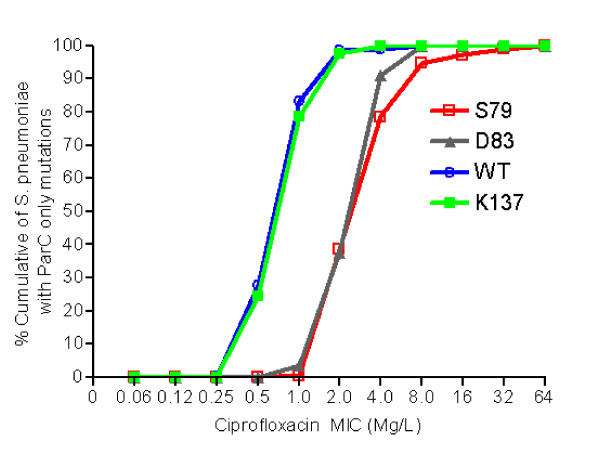
**Distribution of ciprofloxacin MICs in *S. pneumoniae *isolates with a *parC *only amino acid substitutions**. The figure shows that pneumococcal isolates with substitution at either position 79 or 83 of *parC *have four-fold increased ciprofloxacin MIC compared to isolates with no mutations in *parC*. On the other hand, substitution at position 137 of *parC *does not appear to increase ciprofloxacin MIC.

Among isolates with *parC *only mutations, the most common mutations were at positions 137 (Lys-137) (N = 722, 70.0%), 79 (Ser-79) (N = 209, 20.2%), and 83 (Asp-83) (N = 56, 5.4%) (Table 2). The most common substitutions observed were Lys137Asn, Ser79Phe or Tyr, and Asp83Tyr or Asn. As shown in figure 1A, substitutions at both positions 79 (Ser-79) and 83 (Asp-83) were associated with four-fold increase in MIC to ciprofloxacin (P < 0.0001). In contrast, substitution at position 137 (Lys-137) was not associated with increased MIC to ciprofloxacin when compared to isolates with no mutations in *parC *(Figure 1A). These data are consistent with previous studies in which isolates with mutations that resulted in Ser-79 or Asp-83 amino acid changes were associated with decreased susceptibility to the fluoroquinolones [25,26,28,31]. Other mutations were found much less commonly. Those occurring at position 78 (Asp-78), and possibly those at positions 52 (Ser-52), 91 (Asn-91), 115 (Ala-115) and 129 (Tyr-129) could be associated with increase MIC to ciprofloxacin, though statistical analyses were not done due to small number of isolates (Table 2). The presence of Ser-79 and Asp-83 mutations were associated with an approximately two fold increase in levofloxacin and moxifloxacin MICs, though none of the isolates with an amino acid change a position 83, and only 1.6% and 4.3% of isolates with Ser-79 mutations were non-susceptible to moxifloxacin and levofloxacin, respectively (data not shown). However, it is important to note that the classification of non-susceptibility is based on CLSI breakpoints. *S. pneumoniae *can just as readily develop resistance to fluoroquinolones during therapy when a first-step mutation has occurred that results in reduced susceptibility to the fluoroquinolones, even though the increase in MIC does not meet CLSI criteria for non-susceptibility. The combination of two mutations, each of which causes a reduction in the activity of the fluoroquinolone, results in a resistant strain [36,37].

**Table 2:** *In vitro *activity of ciprofloxacin against *S. pneumoniae *with mutations in the *parC *gene only.

		Ciprofloxacin Minimum Inhibitory Concentration (mg/L) Number of Isolates										
**Substitution**		**0.12**	**0.25**	**0.5**	**1**	**2**	**4**	**8**	**16**	**32**	**64**	**Total**

None		1	6	919	1,863	523	37	2	2	0	1	3,354

Asp-78		0	0	0	0	4	2	0	0	0	0	6

Asp-83		0	0	0	2	19	30	5	0	0	0	56

Lys-137		0	1	176	391	137	16	1	0	0	0	722

Arg-95		0	1	2	2	0	1	0	0	0	0	6

Ser-52		0	0	1	3	4	1	0	0	0	0	9

Ser-79		0	0	0	1	82	84	33	5	2	2	209

Other*		0	0	3	6	7	1	0	0	0	0	17

Tyr-129		0	0	0	3	3	1	0	0	0	0	7

*Mutations at other sites include: Ala-63 (1), Val-66 (2), Met-85 (1), Asn-91 (2) Glu-99 (1), Glu-100 (1), Gly-106 (1), Ser-107 (1), Met-108 (1), Ala-115 (2), Tyr-118 (1), Arg-122 (1), Leu-130 (1), Glu-135 (1), Thr-138 (1), Ala-158 (1). Of these, two (one with Asn-91-Asp mutation, and one with Ala-115-Pro mutation) had ciprofloxacin MICs of 4 mg/L, and one (Thr-138-Ile) had a levofloxacin MIC of 2 mg/L. All others had MIC to ciprofloxacin of < 4 mg/L, to levofloxacin of < 2 mg/L, and to moxifloxacin of < 0.5 mg/L)

We found that not all mutations resulted in a reduction of the activity of the fluoroquinolones. An amino acid change at Lys-137 in combination with a change in either Ser-79 or Asp-83did not significantly increased the ciprofloxacin MIC compared to isolates with Ser-79 or Asp-83 only changes (Ser-79 only vs Ser-79 + Lys-137: P = 0.330; Asp-83 only vs Asp-83 + Lys-137: P = 0.5511). These data are consistent with previously reported studies in which a mutation at *parC *resulting in an amino acid change at Lys-137 did not play a significant role in increasing the MIC to fluoroquinolones [30,32].

If the Lys-137 parC mutation has only a marginal effect, if any, on fluoroquinolone activity, what would explain its presence and the frequency with which has been identified by others [38]? Its occurrence may just be the result of a polymorphism in *parC*, similar to what has been seen in *Streptococcus pyogenes *[39] or as yet some unidentified fitness advantage. The frequency with which it has been found is due in part to its association with specific common serotypes. We found that 400 out of 622 (64.3%) isolates with Lys-137 *parC *mutation belonged to serotypes 1, 12, 31, 7A, 9V, 9N and 9L, whereas only 49 out of 3064 (1.6%) of isolates with no mutations belonged to these serotypes. Among these serotypes, 9V, 9N and 9L contained the majority of isolates with Lys-137 *parC *mutation (285 out of 400).

In Canada, there has been a sustained yearly increase in the use of the fluoroquinolones for the treatment of respiratory tract infections in adults during the last decade [6]. This has been accounted for in large part by the increased use of levofloxacin and moxifloxacin [6]. Despite the increased use of fluoroquinolones such as moxifloxacin that preferentially target GyrA, the number of mutations in the QRDR of the *gyrA *have gene remained low [40]. We found only 30 out of 3297 (0.9%) isolates containing mutations in *gyrA *only that resulted in an amino acid change in GyrA, with the most common substitution observed at position 81 (Ser-81) (70%; 21 out of 30) (Data not shown). The most common substitutions observed were Ser81Phe or Tyr in *gyrA*. The presence of a mutation in *gyrA *that resulted in an amino acid change at position 81 was associated with an approximately two fold increase in MIC to ciprofloxacin with 52.4% (11 out of 21) of isolates with this mutation resistant to ciprofloxacin. These data suggest that Ser-81 mutation in *gyrA *significantly reduces susceptibility to all fluoroquinolones.

**Figure 2:**
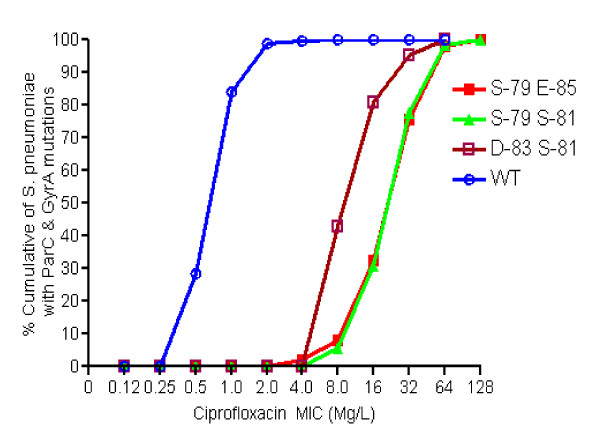
**Distribution of pneumococcal isolates with amino acid substitutions in *parC and gyrA *against ciprofloxacin MIC**. The figure shows that isolates with amino acid substitutions at positions 79 of *parC *and 81 of gyrA or at positions 79 of *parC *and 85 of *gyrA *have 32-fold higher ciprofloxacin MIC compared to isolates with no mutation in *parC *and *gyrA*. Similarly, substitutions at positions 83 of *parC *and 81 of *gyrA *result in 16-fold increase in ciprofloxacin MIC.

Among isolates with mutations in both *parC *and *gyrA*, majority of these isolates carried amino acid alterations at Ser-79 and Ser-81 of *parC *and *gyrA*, respectively (214 of 298; 71.8%), followed by amino acid alterations at Ser-79 and Glu-85 (45 of 298; 15.1%). As shown in figure 2, isolates with mutations Ser-79 in *parC *and Ser-81 in *gyrA *were significantly associated with higher ciprofloxacin MIC with 69% (145 out of 210) isolates had MIC ≥ 32 mg/L. Similarly, isolates containing mutations Ser-79 in *parC *and Glu-85 in *gyrA*, or Asp-83 in *parC *and Ser-81 in *gyrA *were also significantly associated with increased MIC to ciprofloxacin. Interestingly, we had found 4 isolates containing mutations Lys-137 in *parC *and Ser-81 in *gyrA*, all of which were resistant to ciprofloxacin. However, MIC values were similar to the strains with mutations in *gyrA *ser-81 only, supporting the fact that mutation in *parC *Lys-137 is not associated with a reduction in fluoroquinolone activity.

## Conclusion

From this study of a large contemporary collection of pneumococci, we have found that, despite the widespread use of the fluoroquinolones for the treatment of respiratory tract infections in adults that resistance remains less the two percent. Also, despite the increase use of the C-8 methoxy fluoroquinolones including moxifloxacin and gatifloxacin which primarily target the type II topoisomerase GyrA, isolates with only mutations in the QRDRs of the *gyrA *only gene remain infrequent. Not all mutations, most frequently Lys-137, found in the QRDRs of the *parC *gene of *S. pneumoniae *is associated with an increased MIC to fluoroquinolones. The high prevalence of Lys-137 appears to be due to its frequent occurrence in common serotypes.

## Competing interests

SNP, RM and KG have none to declare. AM has served on speaker's bureau, and on advisory boards of Bayer and Wyeth. She has also received unrestricted investigator grants from Bayer and Wyeth. DEL is a member of Advanced Life Sciences and Boehringer Ingelheim USA Corporation advisory board committees. He has also consulted with MPM Asset management LLC., and has received research support from Advanced Life Sciences, Cerexa, Inc, GlaxoSmithKline, Inc., and Bayer Healthcare AG.

## Authors' contributions

SNP carried out statistical analysis of the data and drafted the manuscript. RM sequenced the isolates and helped in drafting the manuscript. AM participated in the design of the study and in the preparation of the manuscript. KG was involved in creation of the database to include all information regarding each isolate. DEL participated in the design and in the coordination of the study, and in the preparation of the manuscript. All authors read and approved the final manuscript.
